# Characteristics of Older Adults Seeking Dementia Prevention Intervention: Insights From the My Healthy Brain Trial

**DOI:** 10.1093/geroni/igaf122.171

**Published:** 2025-12-31

**Authors:** Makenna Law, Christine Ritchie, Olivia Okereke, Bettina Hoeppner, Judson Brewer, Stephen Bartels, Ana-Maria Vranceanu, Ryan Mace

**Affiliations:** Massachusetts General Hospital, Boston, Massachusetts, United States; Massachusetts General Hospital, Boston, Massachusetts, United States; Massachusetts General Hospital, Boston, Massachusetts, United States; Massachusetts General Hospital, Boston, Massachusetts, United States; Brown University, Providence, Rhode Island, United States; Massachusetts General Hospital, Boston, Massachusetts, United States; Massachusetts General Hospital, Boston, Massachusetts, United States; Massachusetts General Hospital, Boston, Massachusetts, United States

## Abstract

Many older adults seek dementia prevention interventions because of heightened concern about cognitive decline, yet little is known about their demographic and clinical characteristics. To address this gap, we examine the baseline presentations and characteristics of older adults with subjective cognitive decline enrolled in a NIA Stage 1B feasibility randomized clinical trial of the virtual My Healthy Brain group mindfulness-based dementia prevention program. We received 406 inquiries, screened 314, and enrolled 62 older adults using a combination of local in-person (senior centers and Boston-area community organizations) and national online (newsletters, registries) recruitment methods. Most participants self-referred (389/406, 95.8%) through flyers, community presentations, online recruitment platforms, and friends. Descriptive statistics were used to characterize the sample according to sociodemographic factors, SCD presentation, physical function, and participation motivators collected at baseline. Participants (M = 71, SD = 6.98) were majority female (81%), white (68%), non-Hispanic or Latino/a (90%) with a family history of dementia (58%). Participants endorsed SCD across multiple domains, with only 15% reporting one domain: mainly memory (69%), language (60%), and attention (47%). Major risk factors for dementia included hypertension (61%), sleep disorders (52%), hypercholesterolemia (35%), and thyroid disease (23%). Participants reported high levels of motivation for behavioral change (92% very motivated), especially regarding physical activity (86%), nutrition (73%), and mental activity (65%). These findings can guide future work by examining associations between baseline characteristics and intervention outcomes. They also emphasize the need to evaluate strategies that promote equity across demographic groups and various stages of dementia risk.

